# Patient experiences with patient-reported outcome measures in metastatic breast cancer trials: qualitative interviews

**DOI:** 10.1186/s41687-022-00460-z

**Published:** 2022-06-03

**Authors:** Carla Romano, Nimanee Harris, Ari Gnanasakthy, Denise D’Alessio, David Chandiwana

**Affiliations:** 1 RTI Health Solutions, 200 Park Offices Drive, Research Triangle Park, NC 27709 USA; 2 Novartis Pharmaceuticals Corporation, East Hanover, NJ USA

**Keywords:** Patient-reported outcome measures, Oncology, Metastatic breast cancer, Semistructured interview, Barriers, Facilitators

## Abstract

**Background:**

Patient-reported outcome (PRO) measures are critical for assessing treatment benefit of anticancer treatments. Although PRO measures have been developed with the intention of capturing patient-centric concepts, a gap exists in understanding the patient experience with these tools. We characterized the experience of patients with metastatic breast cancer (mBC) with PRO measures in an oncology clinical trial setting to determine the importance, relevance, barriers, and facilitators for PRO completion.

**Methods:**

The multicenter, qualitative design included semistructured interviews with 18 women with mBC who completed PRO measures in a clinical trial setting within 1 year of screening. Interviews began with concept elicitation to understand symptom characterization, decision to participate in a clinical trial, pre-trial expectations, and thoughts on study-related questionnaires. Cognitive debriefing was conducted to determine if items in a commonly used PRO instrument were relevant to the patient experience. Qualitative data were analyzed using a constant comparative approach.

**Results:**

Participants described the need for detailed explanation of PRO measures at study start, including information about how the PRO data will likely be used to support drug development. Respondents who felt connected to clinical trial research were more likely to feel as if the measures adequately captured their experiences. Items that were deemed irrelevant or out of date to the patient experience may cause a respondent to feel marginalized and adds to the overall burden of PRO measure completion. Mode of PRO measure administration (electronic/paper) was important to some, but placement of the instrument completion within a study visit appeared to influence respondent willingness to fully engage with the measures. A lack of any type of feedback loop to allow respondents to learn from the captured PRO data was noted as important but missing from the patient experience.

**Conclusions:**

PRO measures need to include content that is relevant to the patient experience over the course of the clinical trial period to be considered meaningful to respondents, and administration techniques also impact engagement. Robust communication strategies that socialize the intent, use, and results of PRO data may enrich the patient experience and support greater adherence to PRO completion in future studies.

## Background

“And in August of 2019 she looked at me and said, there's nothing further we can do, and we need to get you in clinical trials because you need time. And what mother would not say yes. Of course, I want time with my children. One just turned four and one will be two in a few weeks. So, this is all they know mommy as, and if I can just get a few good years with them, that's what I want.” [Participant 6]

Breast cancer is the most common cancer affecting women, comprising 24.5% of new global cancer diagnoses in 2020 [1]. An estimated 42,690 patients will die in the United States (US) from breast cancer in 2020, with the overwhelming majority of these deaths being attributed to metastatic breast cancer (mBC) [2]. Recent improvements in treatments and earlier detection have increased survival rates for women with metastatic breast cancer, although the 5-year survival rate of 26% highlights the need for new advances in treatment [3]. Systemic therapies, including chemotherapy and immunotherapy, are the principal treatments for mBC [4]; however, the trend of declining mortality observed since the 1990s has slowed [2]. Advances in systemic therapies are needed and will require clinical trials to evaluate the efficacy and safety of promising treatments.

Despite the more than 1.6 million cases of breast cancer diagnosed globally each year, a gap remains in the understanding of disease-specific, health-related quality of life (HRQOL) and patient satisfaction with treatments currently available for mBC [5, 6]. There is growing use and acceptance of patient-reported outcome (PRO) measures in trials of mBC to assess both HRQOL as well as key signs and symptoms intended to be specific to both disease and treatment burden from the perspective of the patient [7, 8]. Patient-reported outcome measures are key to the evaluation of treatment benefit of anticancer treatments and provide critical data beyond clinical indices [9], playing a pivotal role in new drug approvals. The use of PRO data is also critical for the computation of quality-adjusted life-years, which are used in health technology assessments [10]. Indeed, the literature has frequently demonstrated the actual patient voice is absent when it comes to the planning, implementation, and use of PRO data in cancer clinical trials [11–13]. An increased focus on collection of patient-experience data, including data from PRO measures, is needed to overcome this gap.

The rigorous capture of patient-experience data is increasingly gaining recognition as an important component of patient-focused drug development (PFDD), which the US Food and Drug Administration (FDA) describes as a development approach to capture and incorporate experiences and perspectives of patients into drug development and evaluation [14]. The FDA further acknowledges that patients have a unique understanding of their disease-related experience and that patients need to be involved in the drug development process [14]. In the oncology setting, expectation for the capture of patient-experience data is evidenced by multiple treatment guidelines from both the FDA and the European Medicines Agency (EMA), as well as by value frameworks proposed by the American Society of Clinical Oncology (ASCO) and the European Society for Medical Oncology (ESMO) [15–20]. However, despite the careful process to identify, select, and develop PRO measures that accurately capture patient expectations, values, and needs, minimal research has been conducted to evaluate the patient experience of interaction with these tools in the context of an oncology clinical trial setting. Research on the patient experience with PRO measures is needed to further contribute to supporting patient-focused drug development.

The objective of this study was to characterize the mBC patient experience with PRO measures in an oncology clinical trial setting to determine the importance, relevance, barriers, and facilitators for completion.

## Methods

### Study design

This multicenter, qualitative study included semistructured interviews with women living with mBC in the US who recently completed PRO measures in a clinical study setting within 1 year of screening. Disease-related symptoms and impacts deemed most meaningful from the patient perspective were elicited to identify specific factors regarding the patient experience most relevant for assessment via standardized questionnaire (i.e., PRO measure). Specific data were collected from participants related to their experience with the administration of PRO measure(s) (i.e., mode of administration, post progression data collection), compliance (i.e., timely and regular completion), training, and relevance of instrument content. Elements of patient burden regarding completion of study-related questionnaires were also assessed. All data were gathered directly from the participant via cognitive interviews, which began with concept elicitation. Concept elicitation seeks to gather spontaneous participant feedback by asking open-ended questions. Following concept elicitation, a brief cognitive debriefing exercise was conducted where a commonly used PRO instrument was assessed by participants to provide characterization of the patient experience. The PRO measure used for cognitive debriefing was a breast cancer–specific HRQOL instrument measuring symptoms and function. The tool asks patients to recall their experiences over the past week. This particular PRO measure was chosen because it is commonly used in cancer trials. However, the focus was not to debrief on the specific tool but instead to gather findings that will be generally applicable across PRO instruments specific to breast cancer. This study was deemed exempt from oversight by the RTI International Institutional Review Board.

### Participant selection

A purposeful sampling approach was taken to ensure enrolled participants experienced the key study concept (i.e., completion of PRO measures in a clinical trial for mBC) [17, 21]. A purposeful sampling approach is in alignment with recent FDA guidance that provides for the use of a purposeful sample to ensure representativeness in exploratory studies focused on the patient experience [17]. Participants with mBC were initially identified and screened through a qualitative research facility and an advocacy group. Individuals interested in participating in semistructured, individual telephone interviews were then contacted by study researchers, who confirmed eligibility using a screener. Respondents meeting inclusion criteria and providing informed consent were scheduled for interview. Eligibility requirements included being ≥ 18 years old and female, having a confirmed diagnosis of mBC within the previous 2 years, having no concurrent diagnoses of other types of cancer, and having participated in at least one clinical trial intended to treat mBC where PRO measures were administered and completed by the participant.

### Interview process

Two qualitative research experts conducted approximately 60-min telephone interviews with patients with mBC using a semistructured interview guide. Use of a semistructured process facilitates a more detailed capture of the patient-lived experience than a completely structured process, and therefore results of semistructured interviews are more truly reflective of what participants consider important and subsequently enrich the data capture [22]. The interviews began with concept elicitation, which gathered spontaneous respondent feedback by asking open-ended questions to prompt participants to discuss their experiences living with mBC. Targeted open-ended questions regarding symptom evolution over time, most bothersome symptoms, and decision to participate in a clinical trial were asked. An example of this approach is illustrated in the following question in which participants are asked to “*please describe any ways having breast cancer has affected your daily life*.” Pre-trial expectations, as well as experiences, were explored and then followed by additional targeted probes to understand participant thoughts on study-related questionnaires. Following the concept elicitation portion of the interview, an abbreviated cognitive debriefing of one HRQOL PRO measure specific to breast cancer was conducted to gather information about patient interpretations of the items and response options. All interviews were audio recorded, transcribed, and quality checked.

### Analytical methods

Qualitative data were analyzed by using a constant comparative approach in which each coded interview was compared with the previous participant’s interview to identify predominant and emergent themes [23]. The output of the qualitative analysis was summarized in terms of thematic outcomes. Two experienced qualitative researchers conducted the analysis of the qualitative data in a standard, systematic manner to ensure the capture of an accurate reflection of the results across all interviews. Results of interviews were reviewed to summarize and identify patterns in the way participants interpreted and responded to each item and to determine how well the items captured concepts relevant to the participants. Quotes representative of participant feedback illustrated key symptoms and patient perceptions of the meaningfulness of questionnaires and clinical trial experience. Quantitative analysis of patient characteristic data included computation of frequencies and percentages for categorical variables (e.g., gender, education level) and the computation of means and ranges for continuous variables (e.g., current age, time since diagnosis).

## Results

A total of 18 adult women with confirmed mBC and previous clinical study experience participated in the study interviews. Participant characteristics and demographic data are presented in Table 1. The average age of the participants was 40.9 years (range 29–68 years), 83% (15/18) were White, and 94% (17/18) had at least some college.

**Table 1:** Characteristics of Participants

Characteristic	(N = 18)
Age, years	
Mean	40.9
Range	29–68
< 40, n (%)	8 (44.4)
40–60, n (%)	9 (50.0)
> 60, n (%)	1 (5.6)
Time since diagnosis, years	
Mean	2
Range	1–4
Race/ethnicity, n (%)	
White	15 (83)
White/Asian	1 (5)
Hispanic	1 (5)
Black	1 (5)
Employment, n (%)	
Full-time	7 (39)
Not employed	7 (39)
Not reported	4 (22)
Education, n (%)	
High school	1 (5)
Some college	15 (83)
College degree	2 (11)

### Concept elicitation

#### Disease and treatment-related signs, symptoms, and impacts

The majority (72%) of study participants described some type of physical, disease-related symptom prior to or at the time of diagnosis (Table 2). These symptoms were varied, but most typically included fatigue or lack of energy, bodily pain including muscle pain, joint pain, cough, headache, gastrointestinal symptoms (e.g., pain, nausea, vomiting, diarrhea), and breast or chest pain. Most attributed these symptoms or sensations to other causes (e.g., too much exercise) but ultimately sought care when symptoms did not resolve over time. The types of signs and symptoms following diagnosis were frequently a continuation or worsening of those previously described, were characteristic of disease progression (e.g., bone metastasis), or were seen as treatment related (e.g., surgical side effects, gastrointestinal symptoms).

**Table 2:** Disease and treatment-related signs, symptoms, and impacts reported at time of diagnosis or following diagnosis

Signs/symptoms /impacts	Participant quotes
*Signs/symptoms/impacts described at diagnosis*	
Fatigue or lack of energy	I was just feeling always tired and, well, run down. [Participant 1]
	… I was starting to, like, really experience were hard breath, I had trouble with my digestive tract, I had some lung issues, I couldn’t breath as well as I generally would like to. And just, I felt overall fatigue. I’m an… I’m a very active person. I am a dance instructor, so I just used to get tired very quickly, frequent urination, things like that. And just overall I didn’t feel as healthy as I usually felt. [Participant 12]
Joint pain (hips, back)	So, I had done a 10-mile charity bike ride with one of my children, and afterwards my back hurt really bad. I wasn’t sure why. [Participant 2]
	I had been having left hip pain probably for at least a month or so prior to…the hip actually fractured. That was kind of what led to the diagnosis. But no other symptoms otherwise. [Participant 13]
Cough	And a few months prior to that I started coughing a lot, and I initially thought it was bronchitis and went to urgent care and was treated for bronchitis [but] didn’t get any better [Participant 4]
Headache	I started getting headaches and this started in the summer of 2017, and I was getting the headaches periodically, not every day. And by winter, by December, actually, it had gone to full-blown headaches. I would wake up in the morning and I would have this headache and it would last literally until I would go to bed. [Participant 5]
No symptoms	I felt good, I felt normal. I was still working my full-time job. So, it was a complete shock to me because I didn’t feel any different. [Participant 3]
	I didn’t really have any symptoms. I was diagnosed with metastatic in August of 2019. I was first diagnosed with stage two triple-negative breast cancer July of 2018, and I had just had a baby in March of 2018. I’d had her and was breastfeeding and found the lump. [Participant 6]
*Continuation or worsening of signs/symptoms/impacts described at diagnosis*	
Chronic fatigue or lack of energy	I feel like my lungs are on fire, like they’re burning is when I do stairs, when I have to do quite a few stairs. [Participant 5]
	I’m still really fatigued. I have a lot of… the cancer, actually. It was spread to my bones when they found it. So, I do have a lot of bone pain, but I am on painkillers. I get multiple joint pains. [Participant 8]
Joint pain (hips, back)	I’ve had pain in my bones. I have pain in my hip; I had to get a hip replacement last year. I have pain in sites where my cancer is. [Participant 3]
*Signs/symptoms/impacts characteristic of disease progression*	
Other	Had an incomplete fracture of my left hip where they…when they discovered that I had a lesion of that in the femur there. So, I have got to have a lovely hip replacement, which had gone well. The biggest thing that I had is to treat, when they did the original diagnosis, I had a, was it a four or a seven? I can't remember the exact size right off hand. I had a four, let's say a four-centimeter mass in the center of my chest that was collapsing my lung and enlarged lymph nodes, so I had…actually I turned out massive amounts of radiation to kill that, and what happened is the bleed off scarred my right lung… so I just have a constant cough and I don't have full function of that lung. [Participant 4]
*Treatment-related signs/symptoms/impacts*	
Surgical side effects (breast pain, lymphoedema)	I wound up having, got a mastectomy, just the one. They didn't do a double. They didn't see anything on the other side, so it's good there. And I had reconstruction done. Was painful… with the chemo and still on medication. [Participant 1]
Gastrointestinal symptoms (nausea, vomiting, diarrhea)	I have a lot of diarrhea symptoms, which are side effects of my medications. And sometimes I get nausea and little bit of vomiting, but it's not consistent. [Participant 2]
Other	So, it's been hard to get used to all of the drugs, and I think I've also become a little paranoid because every time I feel like something's wrong, I just assume that it's disease progression. [Participant 9]

Symptoms caused downstream impacts on patient lives (Table 3). Several participants described the impacts of fatigue on their ability to complete activities of daily living around the home and caring for family, impacts on paid work and productivity, lack of ability to exercise or participate in previously enjoyable hobbies, and lack of ability to be social. Participants described a willingness to deal with medication side effects and inconveniences of treatment regimens in exchange for increased survival rates but clearly noted the value of time.

“But I know that when we go to our doctor's appointments, we generally have waiting time and that seems like a good time to give someone a piece of paper to fill something out or an iPad to fill something out. And they don't take advantage of that time. And then I have to do it on my own time when I don't want to.” [Participant 9]

“I'm 36 and I have two toddlers that I want to be a mother to, so I will do whatever I need to do to prolong that honestly.” [Participant 6]

**Table 3:** Participant quotes describing impacts on patients’ lives

Impacts	Participant quotes
Physical functioning (decreased physical activity, work limitations, household and family limitations)	Oh yes. It's a huge impact. Always tired. Tired and fatigued. I'm limited…like my work, I may work 20 h per week due to doctor's appointments, treatments. And I have a 15-year-old and a 7-year-old, so a lot of things I cannot do because of my health. Simple household stuff is very limited. I probably can stay up on my feet probably about 20 min and then I have to sit down. [Participant 17]
	I own a business and it’s me and my husband together, so it’s impacted me and the responsibility that I have to do, but it’s not that I’ve had to go out on disability or things like that. I’ve still been able to kind of help and direct. He’s picked up a lot of the pieces since all of this has happened. I’m not doing my usual activities like yoga, things that really require a lot of energy or taking trips and drinking that might be adventurous and taking trails and hikes. [Participant 15]
	So, for me, the symptoms are more around feeling tired, not being able to exercise as much as I would like to. I can't take long walks. I used to be a runner. I can't do that anymore. I mean I can, but I just get really, really tired quickly. [Participant 12]
	Definitely when my tiredness was getting worse and I was always run down. And I just felt… I felt like I had the flu, and what I kept telling people. And, yeah. And it felt, I couldn't get out. [Participant 1]
Social life and motivation	It's not fun. Sometimes it's really hard to get up in the mornings, to get going, because my body's sluggish and I'm tired. It was hard, like when I was working, to get up and get going. And getting to work and being there all day, it was draining. So, it's definitely affected my life and my social life, and what me and my husband used to like to do. Now it's like, it's not enjoyable for me anymore. [Participant 3]
Importance of time as a concept	I'd be then I'd be okay what are the side effects is that four months’ worth that amount of side effects. That's what I would be considering. Yeah, but time, time is, I mean, my first key… [Participant 2]
	I'm 36 and I have two toddlers that I want to be a mother to, so I will do whatever I need to do to prolong that honestly. [Participant 6]
	Haven't had surgery. Like I said originally, I thought I would maybe do reconstruction, but at this point, it's not worth my time being holed up in recliner when I can… [Participant 6]

#### Clinical trial experiences

Participants elected to participate in a clinical trial for varied reasons; however, nearly half (45%) made the decision based on a recommendation from their oncologist (Table 4). Others described being self-motivated to search for research opportunities while indicating they wished to be as aggressive as possible in treating their mBC. All patients sought additional quality survival time and hoped a trial would offer this option. One participant participated in a noninterventional study that only involved completing questionnaires based on a request to her while in the hospital; all other participants were included in interventional clinical studies.

"They know what the progression is, and it's very, very fast, and pretty ugly, that's what it is. So I had to make a decision about a clinical trial, and it was double-blinded, which was like... wasn't prepared to do that. But as a result of that, I'm doing really, really well, so I'm sort of looking at it as I got my Hail Mary, and I got my miracle for as long as it lasts." [Participant 7]

Most respondents felt they were aware of the underlying purpose for the PRO measure data collection, but a few noted a lack of an informative overview for questionnaire completion. In these cases, the questionnaires had been introduced as “self-explanatory” and respondents were to simply expect them in the mail or email (during the clinical trials for which they participated). Those who did receive an introduction noted the study coordinators or study nurses thought questionnaires would be useful to evaluate the study drug. Additionally, the level of instruction for completion also varied, with some study sites providing detailed background and instructions for PRO measure collection while others took a more hands-off approach. Importantly, the former approach appeared to influence respondent perceptions of the questionnaires and the importance of the questionnaire data. While a hands-off/self-explanatory approach could be considered less burdensome from a patient perspective, as it could shorten a study visit, it was also perceived by some as a potential barrier. Without the explanation of the purpose behind the data collection and review of measures (at least at the initiation of the questionnaires), subjects were not provided support or an opportunity to ask questions regarding the materials.

“They just said, 'Expect these in the email.' And it was about... I think they ended up being full of... I want to say five to six pages. They were kind of lengthy pages, and so cumbersome, but, again, I only got them once a month, probably, so it wasn't terrible. But didn't give me information prior to it.” [Participant 1]

“So when I went in, one of their representatives actually sat and walked me through the entire process, made sure I was comfortable. Sort of reassured me about everything and there were a lot of different forms and consent forms that I was also supposed to fill out.” [Participant 12]

Participants were asked to reflect on how meaningful they found the content of the PRO questionnaires included in the clinical trials. Overall, responses supported inclusion of the PRO measures based on the instrument concepts, and the goal of PRO data collection was recognized. Participants who reported a positive experience regarding the use of PRO measures were able to reflect on specific concepts within the PRO measure and the relationship between the concept and their own experience. One participant articulated the experience of completing PRO measures as providing an additional connection to the site staff to better describe her situation and to use this as an opportunity to communicate important and relevant information. However, interview respondents had varied views on the content of the PRO measures depending on the topic, introduction to the questionnaires, and administration schedule. Some respondents commented that they felt “annoyed” by the repetitive nature of questions on a questionnaire or the frequency of follow-up administrations of the same questionnaire, indicating that excessive repetition of questions or administration, as well as delays in administering questionnaires while the participant is at the site, could be additional barriers to fully engaging in PRO completion in a meaningful manner.

“And instead of the 10 or 15 minutes that they generally allot to me with the nurse practitioner, I was probably in there, like, 40 minutes and we discussed everything.” [Participant 4]

It was constant: are you having nausea, diarrhea, vomiting, do you have this pain, do you have this? It was just endless, to the point to be honest with you I just started marking it ‘No, no, no, no.’ It was just so repetitive that it got to being annoying. [Participant 15]

“I felt it was relentless, it was just the same thing over and over; repetitive…I'm here today and I'm here next Friday. I'm in the same situation. I'm not any different. I wish I could've said, 'Hi I'm here...[name deleted]...and I'm the same,' and go, versus having to stay and sit and fill paperwork out. It was just...that's why I wanted to drop out. I was just tired and exhausted; not enjoyable.” [Participant 18]

**Table 4:** Reasons for patients’ participation in clinical trials and understanding of the purpose/content of PRO questionnaires

	Participant quotes
Reasons for participation	And in August of 2019 she looked at me and said, there's nothing further we can do, and we need to get you in clinical trials because you need time. And what mother would not say yes. Of course, I want time with my children. One just turned four and one will be two in a few weeks. So, this is all they know mommy as, and if I can just get a few good years with them, that's what I want. [Participant 6]
	Well, my oncologist, she's been with me since the beginning of my stage two, so she actually was the one who recommended the trial. And she's, like, look let's try this out first and she's, like, I've seen good… I've seen it work on some patients and others not. She's, like, let's give this a try and so, luckily, thank God it's been working on me. [Participant 5]
	Well for one thing I just wanted to take, like, the most aggressive approach that I could take, so I was, like, kind of looking at clinical trials from the moment I was diagnosed with metastatic disease. Because I know that is like the forefront of treatment and I really wanted to be aggressive. [Participant 16]
Purpose and content of study-related questionnaires	I knew there were going to be questionnaires. I don't feel like they were explained. I feel like they were pretty self-explanatory themselves. If I had questions, there was always somebody available that I could reach out to. [Participant 13]
	So, when I went in, one of their representatives actually sat and walked me through the entire process, made sure I was comfortable. Sort of reassured me about everything and there were a lot of different forms and consent forms that I was also supposed to fill out. [Participant 12]
	I mean, we know it was research, like when they told us about it, there was a large population of who… of how many people… or how many participants were in it and why they were gathering research, to help and for that data, and specifically look and analyze the side effects and cost versus benefit, and how certain other meds can help, and how the process, and help get educated on by your doctors [Participant 1]

#### Meaningfulness of study-related questionnaires

Responses seemed dependent upon how well the respondent understood the purpose, importance, and intent of the questionnaires in the clinical trials. Some felt the questions did not adequately capture the full impact of mBC and therefore did not adequately assess disease burden or treatment benefit. While most respondents recognized the importance of the PRO data to inform clinical trial findings, a third (33%) felt the data could have been better optimized. Specifically, several respondents noted the lack of a “feedback loop,” indicating that they sometimes felt their information/PRO data was going into "thin air" and would have preferred to receive information back about the study findings.

"But I submit it into thin air. And it makes me feel like it doesn't matter. I could circle whatever numbers I want, it doesn't really matter. And I don't do that but I, sometimes when you're kind of between a little bit or very much and you actually think about it, I wouldn't take the time to think about it, I would just pick one and move on. Because I don't, in my head I believe someone should be following up with me and then they don't and they're just...

But it was nice if at the end of it there's something that's created and you shared it with the participants, they really, that make people feel that they were valued and their opinions were valuable…

But being a patient, answering these questions, it makes me feel like, if I answered the most extreme answers on every single pain question, every single question, my natural expectation would be that there would be a feedback loop where I would then get a call from my doctor to say, let's talk about this, it's so extreme.” [Participant 9]

Notably, age of the respondent was also a key factor regarding relevancy of a PRO used in a trial setting (Table 5). One woman articulated this, noting that less than half of the items in the PRO questionnaires included in the clinical trial were reflective of her life situation given her young age of 29 years. Administration schedules and mode of administration also seemed to impact participant perception of PROs as described by half of respondents (50%). For example, concerns about meaningfulness of repeated static items, or time/space conditions for completing PRO measures all appeared to influence participants’ perceptions. Some respondents described the use of electronic data collection for PROs in a clinical trial to support the ease of use, while some others were more comfortable with paper.

“…factor of age plays in. I'm 29, so I was pretty healthy before I was diagnosed and I was straight-up diagnosed with metastatic. So for me, the symptoms are more around feeling tired, not being able to exercise as much as I would like to. I can't take long walks. I used to be a runner. I can't do that anymore. I mean I can, but I just get really, really tired quickly. So they have like around, I would say 50 questions out of which 20 I feel are really relevant to my situation” [Participant 12]

“Yeah, the answer is they're not actionable. They don't matter. And I don't matter. I'm a Jane Doe. And that's how it is, but it doesn't have to be that way because you can just provide consent forms for the information to be transmitted to both as part of the research study." [Participant 9]

**Table 5:** Meaningfulness of clinical trial–related questionnaires to participants and their understanding of PRO data

	Participant quotes
Meaningfulness of study-related questionnaires	I think they did an excellent job. And the study I'm in now here at home, same thing with the questionnaires. I think they're doing a really good job in capturing a lot of the symptoms, and asking the good questions to figure out and try to pull some more information out of me that I don't remember day-to-day that had actually happened. [Participant 3]
	Meaningful not in a positive way, but I found myself very anxious and full of anxiety. I was uncertain and uneasy and a little aggressive; irritable. [Participant 18]
	No. There's one question on there, for example, on mine that says, Do you feel like a woman? I'm like seriously, what does that mean? That is by far the worst question and it's a scale question. Not all, little bit, same kind of thing with very much, I'm like what? The last I checked that didn't change. [Participant 2]
	I think electronic is a lot better. It's paper and pen and circling where, to be honest with you thinking back, pen and paper takes more energy for someone to circle and an electronic version is easier to just click, click, click… [Participant 15]
Use of PRO data	I understand that it's a clinical trial, and I wish people were more straightforward about these things. There may be some questions that you need to ask in a certain way because of how a trial is designed, but there can be a way that you ask questions that make the patient feel like you want to hear from me. [Participant 7]
	My way would be with meeting with the doctor, and I would like the doctor to be the person who is caring for me who has been with me since I was diagnosed. I don't want to have to change to a different doctor, different nurses. I would like it to remain the same… And you know, he knows me. He knows how I am. He knows my history. He can compare…the other people that see me don't know anything and that's why I feel like I'm a statistic…. [Participant 18]

**Fig. 1:**
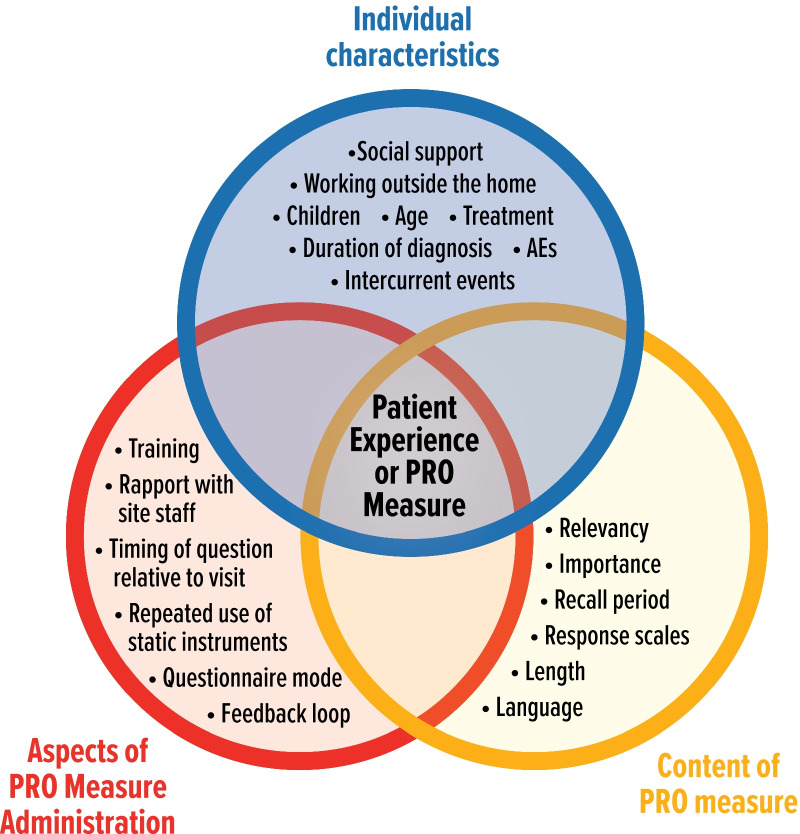
Factors of external validity and meaningfulness of the patient experience with PRO measures. *AE* adverse event, *PRO* patient-reported outcome

Figure 1 displays the hypothesized relationship between external validity factors and meaningfulness of experiences derived from this research.

### Cognitive debriefing

Two individuals were not able to participate in the full cognitive debriefing due to their health status. Participants in the cognitive debriefing (n = 16) generally found the PRO measure to be acceptable in terms of content and format. The instructions were described as clear and easy to understand, the recall period reasonable. Few concepts were noted as missing, and the tool was deemed to be fairly comprehensive. However, relevancy of certain items was strongly dependent upon respondent experience with different types of therapies. Participants indicated some items were relevant only for those who had undergone surgery, others for chemotherapy; additionally, some items may be limited for patients having treatment with immunotherapies. This may be most important for patients mBC as compared to earlier stages where treatment exposures are more limited. A few participants also indicated stage in life might influence responses (e.g., younger vs. older participants). Additionally, time since treatment appeared to influence respondents’ view on the relevancy of particular items. For example, the question “have you lost hair” over the last week could have a potentially misleading response if a respondent had already lost all of their hair.

“...say they're younger, and they're being...or they end up being forced into menopause, and they're having these mood swings, that is a natural side effect of menopause. But you don't expect to deal with it, say, if you're in your twenties or thirties.” [Participant 2]

“I had a surgery 2 years and now I'm not happy with it. That's kind of irrelevant and a little odd [not going to change over the course of her current trial]” [Participant 18]

The order of the questions also appeared to influence respondent feedback. Participants commented on the inclusion of symptom items and items gathering other more complex issues within the same section of the questionnaire. In areas where related questions were separated, respondents thought it would be more useful to group by topic. Regarding missing concepts, two respondents felt there should be questions about hormonal imbalances, while another noted the importance of the free-text items to allow inclusion of any important concepts that were missed.

“I mean the order seems a little funny but... It's kind of all of a sudden… It's, there's symptom after symptom and then it's emotional and then a smaller question about, it does seem a little bit non-sequential.” [Participant 16]

## Discussion

The purpose of this research was to characterize the patient experience with PRO measures in an oncology clinical trial setting to determine the importance, relevance, and barriers to PRO completion, as well as to assess how meaningful participants found the experience. The study results suggested that comprehensive PRO tools and strategies to incorporate measures into the clinical trial setting are needed to effectively characterize the patient journey. Additionally, participants described the need for an understanding of PRO measures at a study outset that includes background information on how the measures will be used to support drug development, need for use of repeated static items, as well as an understanding of the importance of completing the PRO questionnaires. Notably, an understanding of how the PRO data would be utilized and a feedback loop to allow participants to learn from the data captured by the PRO measures appeared to be missing for most participants in the oncology trial setting. The sharing of clinical trial results with participants can be controversial and raises ethical concerns, particularly for those participants who experience psychological stress when a treatment is revealed to be suboptimum [24]. While a near unanimous majority (≥ 95%) of both clinicians and patients in a survey have indicated support for providing participants a summary of trial results, there was broad disagreement between groups and within groups about the preferred timing of the feedback (e.g., after the trial has closed, after participants have completed the trial) and the preferred method of feedback (e.g., results are shared by the participants’ clinicians, shared by the research team, posted online, emailed) [25]. Additional research is needed to understand how to optimally share trial results, including PRO data, with participants in a way that is meaningful for most participants while avoiding harm through psychological stress or the inadvertent release of confidential information. The use of PRO measures in clinical practice where the information is shared back into individual patient care may provide insight in how to approach mitigating these potential harms.

Key factors of the participants’ experience included providing details on how to tie the collection of the data with the use of the data and offering some type of feedback loop, which could include potential publication/reports to study participants. Participants who felt connected to the clinical research and that they were heard by study staff described a greater appreciation of the PROs and were more likely to feel as if the PROs captured their experiences. Participants who felt the process of completing PROs was impersonal or unimportant to study staff did not share this positive feeling and often felt as though they had no real understanding of how their PRO data could be used. Based on study results, it appears that PRO instruments need to include content that is clearly and closely relevant to the patient experience over the course of the clinical trial period in order to be considered meaningful to study participants.

These findings support that particular care must be taken to identify or develop instruments that are fit for purpose and therefore relevant to patients matching the inclusion/exclusion criteria within the trial (for example, age, treatment history) in order for PRO measures to be most successful in accurately capturing the patient voice. Patient-experience data collected using PRO measures can help researchers understand certain aspects of mBC and its treatment; however, if the face validity of PRO items is not readily apparent to the respondent, effort must be made to explain the importance of collecting the PRO data. Aspects of PRO measure administration, including the timing of questions relative to visit and rapport with site staff, were raised as barriers by participants. However, these same aspects of PRO measure administration could be viewed as facilitators to PRO completion when site staff prioritized the time of participants. Indeed, study participants described time as being far too precious of a commodity to feel wasted, indicating time spent on irrelevant questions would not be time well spent. Additionally, participants noted during the cognitive debriefing that existing breast cancer measures may not be specific enough for use in women with mBC, especially with emerging new therapies, as these patients may have experienced a wide range of therapeutic interventions that patients with an earlier stage disease would not. For example, questions that may be fully relevant to a participant with a metastatic diagnosis as a progression of disease may be irrelevant or somehow less important over time (i.e., greater potential for a response shift for such items if these are even able to be accurately scored) to a woman who had mBC as an initial diagnosis.

The life situations of participants were also a key factor in determining the relevancy of PROs to individuals. Women with advanced breast cancer face impacts to their emotional well-being [26]; however, the focus of these impacts can be highly unique to the individuals. In our study, a mother described the motivation for her participation in a clinical trial as the potential to extend her life and therefore allow her more time to spend with her two very young children. However, pregnant women with advanced breast cancer may be primarily motivated by other factors, including fear for their child’s survival [27]. Participants also noted that older patients with mBC, or those at different life stages, face other challenges and impacts to their lives. Exit interviews with patients in a clinical trial, may offer an additional outlet to capture patient-experience data missing from standard PRO measures and allow trial participants to describe disease burden and treatment benefit more fully in a manner that may appear to better recognize the patient as an individual. Incorporating this knowledge into the development of cancer treatments will also promote patient autonomy and agency, which have been identified as being especially important to women with breast cancer [28, 29]. Providing a mechanism for feedback to and from the patient around the collection of PRO data as a standard practice would be extremely beneficial. This feedback should ideally include how PRO data matter and how it led to something “actionable”—either in the trial participant experience or as support for approval of a new treatment.

A limitation of this study is that only participants from the US and those who identified as women were included, with the resulting sample being majority White and female. Additionally, the relatively young age of the study population (almost all participants were aged less than 60 years) did not allow us to capture the experiences of patients over the age of 60 years with breast cancer. Another limitation is that qualitative research may not typically be generalizable beyond an mBC population, especially given that these participants volunteered to be included in the study. However, the results of this qualitative study are both representative and reliable [30]. A purposeful sampling approach contributed to the representativeness of the research in alignment with FDA guidance [17]. A brief cognitive debriefing was conducted to capture the patients understanding of the PRO measure rather than to support instrument modification, although not all participants completed this exercise (16 of 18).

## Conclusions

It is not enough to capture the patient voice if those speaking do not feel heard. The process of collecting the patient voice in the oncology clinical trial setting is facilitated by the creation and use of well-developed, fit-for-purpose PRO measures, robust training for participants, utilization of participant-preferred mode of administration, administration schedules with strong site-level support, and the appropriate, timely dissemination of PRO results to participants. Communication strategies that incorporate a feedback loop to disseminate results of PRO data back to study participants, such as including the data in the plain language summary of clinical trial findings required by the European Medicines Agency, may provide a connection between the participants and the research outcomes that supports greater adherence and continued interest in PRO completion for future studies.

## Data Availability

There are no data sets to share, as data are fully qualitative in the form of transcripts.

## Abbreviations

ASCO: American society of clinical oncology
EMA: European medicines agency
ESMO: European society for medical oncology
FDA: US food and drug administration
HRQOL: Health-related quality of life
mBC: Metastatic breast cancer
PFDD: Patient-focused drug development
PRO: Patient-reported outcome
US: United States
